# Estimation of population mean in the presence of measurement error and non response under stratified random sampling

**DOI:** 10.1371/journal.pone.0191572

**Published:** 2018-02-05

**Authors:** Erum Zahid, Javid Shabbir

**Affiliations:** Department of Statistics, Quaid-i-Azam University, Islamabad, Pakistan; Universitat Zurich, SWITZERLAND

## Abstract

In the present paper we propose an improved class of estimators in the presence of measurement error and non-response under stratified random sampling for estimating the finite population mean. The theoretical and numerical studies reveal that the proposed class of estimators performs better than other existing estimators.

## Introduction

In survey sampling, usually it is assumed that all the observations are correctly measured on the characteristics under study. But in practice this assumption is not met for a variety of reasons, such as non-response may occurs due to refusal of respondents to give the information or not at home or lack of interest or due to some ethical reasons. Usually measurement error and non-response are studied separately using the known auxiliary or additional information. In reality, both measurement error and non-response occur simultaneously in survey sampling. Mostly the information is not obtained from all the units during surveys, so non-response is a common problem which may creeps during a sample survey. In sampling theory the estimation of population mean of a variable of interest in the presence of non-response, when the auxiliary information available is widely debated. [1–5] and [6] discussed the problems of non-response in detail. To estimate the population mean, the researchers dealt with the problem of measurement error. For more details, see [7–11], etc. Recently few researchers studied the problem of measurement error and non-response together like [12–14] and [15]. [16] and [17] studied the improved estimation of population mean in simple and stratified random sampling.

In practice, the researchers who have studied measurement error have ignored the presence of non-response. But very few of them studied both under simple random sampling. In this paper, we have proposed a class of estimators for estimating the population mean in the presence of measurement error and non-response simultaneously under stratified random sampling. The efficiency of the suggested class of estimators over the existing estimators is shown through simulation study and real data sets.

Consider a finite population of *N* identifiable units which are partitioned into *L* homogeneous subgroups called strata, such that the *h*^*th*^ strata consist of *N*_*h*_ units, where *h* = 1, 2, …, *L* and $\sum_{h=1}^{L}N_{h}=N$. It is assumed that *N* consists of two mutually exclusive groups called response and non-response groups. Let *N*_1*h*_ and *N*_2*h*_ are the responding and non-responding unit in the *h*^*th*^ stratum respectively. We select a sample of size *n*_*h*_ from *N*_*h*_ by using simple random sampling without replacement (SRSWOR) and assume that *n*_1*h*_ units respond and *n*_2*h*_ units do not respond. We select a sub-sample of size *k*_*h*_, $\left(k_{h}=\frac{n_{2h}}{g_{h}},g_{h}>1\right)$ from *n*_2*h*_ non-responding units in the *h*^*th*^ stratum.

Let $\left(x_{hi}^{*},y_{hi}^{*}\right)$ be the observed values and $\left(X_{hi}^{*},Y_{hi}^{*}\right)$ be the actual values on the variables (*X*, *Y*) of the *i*^*th*^(*i* = 1, 2, …, *n*) sample units in the *h*^*th*^ stratum. Then the measurement errors be $V_{hi}^{*}=x_{hi}^{*}-X_{hi}^{*}$ and $U_{hi}^{*}=y_{hi}^{*}-Y_{hi}^{*}$. Let $S_{hY}^{2}$ and $S_{hX}^{2}$ be the population variances for the responding units, and $S_{hY(2)}^{2}$ and $S_{hX(2)}^{2}$ be the population variances for non-responding units. Let $S_{hV}^{2}$ and $S_{hU}^{2}$ be the population variances associated with the measurement error for responding units. Let $S_{hV(2)}^{2}$ and $S_{hU(2)}^{2}$ be the population variances associated with measurement error for the non responding part of the population. Further let *C*_*hY*_ and *C*_*hX*_ be the coefficient of variations for the respondents and *C*_*hY*(2)_ and *C*_*hX*(2)_ be the coefficient of variations for the non-responding units respectively. Let *ρ*_*hYX*_ and *ρ*_*hYX*(2)_ be the population correlation coefficients between their respective subscripts respectively for responding and non-responding units, respectively.

In this paper an improved class of estimators for estimating the population mean in the presence of measurement error and non-response is proposed under stratified random sampling. Expressions for the bias and mean square error (MSE) of the class of estimators are obtained upto first order of approximation, when both the study and the auxiliary variables suffer with a problem of non-response and measurement errors.

The present paper is organized as: Section 2 gives some existing estimators of the finite population mean. In Section 3, an improved class of estimators is suggested for estimating the finite population mean by incorporating both measurement error and non-response information simultaneously. Efficiency comparison is presented in section 4. Numerical results and simulation study are presented in Section 5. Conclusion is given in Section 6.

## Existing estimators in literature

In this section we consider the following existing estimators.

### Hansen and Hurwitz (1946) estimator

In stratified random sampling, the Hansen and Hurwitz (1946) estimator for population mean $\bar{Y}$, is given by

$$\begin{array}{c} {\bar{y}}_{S(HH)}^{*}=\sum_{h=1}^{L}P_{h}{\bar{y}}_{h}^{*}, \end{array}$$ (1)

where ${\bar{y}}_{h}^{*}=\left(\frac{n_{1h}}{n_{h}}\right){\bar{y}}_{1h}+\left(\frac{n_{2h}}{n_{h}}\right)\bar{y}\prime_{2h}$ and $P_{h}=\frac{N_{h}}{N}$.

Here ${\bar{y}}_{n_{1h}}$ and $\bar{y}\prime_{2h}$ are the sample means based on *n*_1*h*_ of responding and *g*_*h*_ units of sub-samples from *n*_2*h*_ non-responding groups, respectively.

The variance of ${\bar{y}}_{S(HH)}^{*}$, is given by

$$\begin{array}{c} Var\left({\bar{y}}_{S(HH)}^{*}\right)=\sum_{h=1}^{L}P_{h}^{2}A_{h}, \end{array}$$ (2)

where $A_{h}=\lambda_{2h}\left(S_{hY}^{2}+S_{hU}^{2}\right)+\theta_{h}\left(S_{hY(2)}^{2}+S_{hU(2)}^{2}\right),$ $\theta_{h}=\frac{P_{2h}\left(g_{h}-1\right)}{n_{h}}$, $P_{2h}=\frac{N_{2h}}{N_{h}}$, $\lambda_{2h}=\left(n_{h}^{-1}-N_{h}^{-1}\right)$.

### Ratio estimator

The usual ratio estimator under stratified random sampling, is given by

$$\begin{array}{c} {\bar{y}}_{S(R)}^{*}=\sum_{h=1}^{L}P_{h}\frac{{\bar{y}}_{h}^{*}}{{\bar{x}}_{h}^{*}}{\bar{X}}_{h}, \end{array}$$ (3)

where ${\bar{X}}_{h}$ is known and ${\bar{x}}_{h}^{*}$ is given in Eq (13). The bias and mean square error of ${\bar{y}}_{S(R)}^{*}$, are given by

$$\begin{array}{c} B\left({\bar{y}}_{S(R)}^{*}\right)≅\sum_{h=1}^{L}\frac{P_{h}}{{\bar{X}}_{h}}\left[R_{h}B_{h}-C_{h}\right] \end{array}$$ (4)

and

$$\begin{array}{c} MSE\left({\bar{y}}_{S(R)}^{*}\right)≅\sum_{h=1}^{L}P_{h}^{2}\left[A_{h}+R_{h}^{2}B_{h}-2R_{h}C_{h}\right], \end{array}$$ (5)

where $R_{h}=\frac{{\bar{Y}}_{h}}{{\bar{X}}_{h}}$, $B_{h}=\lambda_{2h}\left(S_{hX}^{2}+S_{hV}^{2}\right)+\theta_{h}\left(S_{hX(2)}^{2}+S_{hV(2)}^{2}\right),$ *C*_*h*_ = λ_2*h*_ *ρ*_*hYX*_ *S*_*hY*_ *S*_*hX*_ + *θ*_*h*_ *ρ*_*hYX*(2)_ *S*_*hY*(2)_ *S*_*hX*(2)_.

### Difference estimator

The usual difference estimator under stratified random sampling, is given by

$$\begin{array}{c} {\bar{y}}_{S(D)}^{*}=\sum_{h=1}^{L}P_{h}\left[{\bar{y}}_{h}^{*}+d_{h}\left({\bar{X}}_{h}-{\bar{x}}_{h}^{{}^{\prime }*}\right)\right], \end{array}$$ (6)

where ${\bar{x}}_{h}^{{}^{\prime }*}=\frac{N_{h}{\bar{X}}_{h}-n_{h}{\bar{x}}_{h}^{*}}{N_{h}-n_{h}}$ and *d*_*h*_ is the constant.

The minimum variance of ${\bar{y}}_{S(D)}^{*}$ is given by

$$\begin{array}{c} Var{\left({\bar{y}}_{S(D)}^{*}\right)}_{min}=\sum_{h=1}^{L}{P_{h}}^{2}\left[A_{h}-\frac{C_{h}^{2}}{B_{h}}\right], \end{array}$$ (7)

The optimum value of *d*_*h*_ is $d_{h(opt)}=-\frac{C_{h}}{t_{h}B_{h}}$, where $t_{h}=\frac{nh}{N_{h}-n_{h}}$.

### Azeem and Hanif (2017) estimator

Azeem and Hanif (2016) estimator under stratified random sampling, is given by

$$\begin{array}{c} {\bar{y}}_{S(AH)}^{*}=\sum_{h=1}^{L}P_{h}{\bar{y}}_{h}^{*}\left(\frac{{\bar{x}}_{h}^{{}^{\prime }*}}{{\bar{X}}_{h}}\right)\left(\frac{{\bar{x}}_{h}^{{}^{\prime }*}}{{\bar{x}}_{h}^{*}}\right). \end{array}$$ (8)

The bias and MSE of ${\bar{y}}_{S(AH)}^{*}$, are given by

$$\begin{array}{c} B\left({\bar{y}}_{S(AH)}^{*}\right)≅\sum_{h=1}^{L}\frac{P_{h}}{{\bar{X}}_{h}}\left[t_{h}^{2}R_{h}B_{h}-q_{h}C_{h}\right] \end{array}$$ (9)

and

$$\begin{array}{c} MSE\left({\bar{y}}_{S(AH)}^{*}\right))≅\sum_{h=1}^{L}P_{h}^{2}\left[A_{h}+q_{h}^{2}R_{h}^{2}B_{h}-2q_{h}R_{h}C_{h}\right], \end{array}$$ (10)

where $q_{h}=\frac{N_{h}+nh}{N_{h}-n_{h}}$.

## The suggested estimator

We propose an improved general class of estimators for estimating the population mean, dealing with the problem of measurement error and non-response simultaneously in stratified random sampling. Measurement error and non-response is present on both, the study and the auxiliary variables. The suggested estimator is given by

$$\begin{array}{c} {\bar{y}}_{S(P)}^{*}=\sum_{h=1}^{L}P_{h}\left[\left\{m_{1h}{\bar{y}}_{h}^{*}+m_{2h}\left({\bar{X}}_{h}-{\bar{x}}_{h}^{{}^{\prime }*}\right)\right\}{\left\{\frac{{\bar{X}}_{h}}{{\bar{x}}_{h}^{{}^{\prime }*}}\right\}}^{\alpha_{h}}exp\left(1-\alpha_{h}\right)\left(\frac{{\bar{X}}_{h}-{\bar{x}}_{h}^{{}^{\prime }*}}{{\bar{X}}_{h}+{\bar{x}}_{h}^{{}^{\prime }*}}\right)\right], \end{array}$$ (11)

where, *m*_1*h*_ and *m*_2*h*_ are constants whose values are to be determined and *α*_*h*_ is the scalar, chosen arbitrary. For obtaining the bias and mean square error, we assume that $\delta_{hY}^{*}=\sum_{i=1}^{n_{h}}\left(Y_{hi}^{*}-{\bar{Y}}_{h}\right)$, $\delta_{hU}^{*}=\sum_{i=1}^{n_{h}}U_{hi}^{*}$.

$\delta_{hX}^{*}=\sum_{i=1}^{nh}\left(X_{hi}^{*}-{\bar{X}}_{h}\right)$, $\delta_{hV}^{*}=\sum_{i=1}^{n_{h}}V_{hi}^{*}$.

Adding $\delta_{hY}^{*}$ and $\delta_{hU}^{*}$, we get $\delta_{hY}^{*}+\delta_{hU}^{*}=\sum_{i=1}^{n_{h}}\left(Y_{hi}^{*}-{\bar{Y}}_{h}\right)+\sum_{i=1}^{n_{h}}U_{hi}^{*}$.

Dividing both sides by *n*_*h*_, and then simplifying, we get

$$\begin{array}{c} {\bar{y}}_{h}^{*}={\bar{Y}}_{h}+\frac{1}{n_{h}}\left(\delta_{hY}^{*}+\delta_{hU}^{*}\right). \end{array}$$ (12)

Similarly, we can get

$$\begin{array}{c} {\bar{x}}_{h}^{*}={\bar{X}}_{h}+\frac{1}{n_{h}}\left(\delta_{hX}^{*}+\delta_{hV}^{*}\right). \end{array}$$ (13)

Further $E{\left(\frac{\delta_{hY}^{*}+\delta_{hU}^{*}}{n_{h}}\right)}^{2}=\lambda_{2h}\left(S_{hY}^{2}+S_{hU}^{2}\right)+\theta_{h}\left(S_{hY(2)}^{2}+S_{hU(2)}^{2}\right)=A_{h},$ $E{\left(\frac{\delta_{hX}^{*}+\delta_{hV}^{*}}{n_{h}}\right)}^{2}=\lambda_{2h}\left(S_{hX}^{2}+S_{hV}^{2}\right)+\theta_{h}\left(S_{hX(2)}^{2}+S_{hV(2)}^{2}\right)=B_{h},$ $E\left(\frac{\delta_{hY}^{*}+\delta_{hU}^{*}}{n_{h}}\right)\left(\frac{\delta_{hX}^{*}+\delta_{hV}^{*}}{n_{h}}\right)=\lambda_{2h}\rho_{hYX}S_{hY}S_{hX}+\theta_{h}\rho_{hYX(2)}S_{hY(2)}S_{hX(2)}=C_{h}.$

On simplifying, we get

$$\begin{array}{ccc} {\bar{y}}_{S(P)}^{*} & = & \sum_{h=1}^{L}P_{h}[\left\{m_{1h}\left({\bar{Y}}_{h}+W_{hY}\right)+m_{2h}\left(t_{h}W_{hX}\right)\right\}(1+\frac{\alpha_{h}t_{h}W_{hX}}{{\bar{X}}_{h}}\\ & & +\frac{\alpha_{h}\left(\alpha_{h}+1\right)t_{h}^{2}W_{hX}^{2}}{2{\bar{X}}_{h}^{2}})exp\left(1-\alpha_{h}\right)\left(\frac{t_{h}W_{hX}}{2{\bar{X}}_{h}}+\frac{t_{h}^{2}W_{hX}^{2}}{4{\bar{X}}_{h}^{2}}\right)], \end{array}$$ (14)

where $W_{hY}=\frac{\delta_{hY}^{*}+\delta_{hU}^{*}}{n_{h}}$ and $W_{hX}=\frac{\delta_{hX}^{*}+\delta_{hV}^{*}}{n_{h}}$.

Further simplifying, and ignoring error terms greater than two, we have

$$\begin{array}{ccc} {\bar{y}}_{S(P)}^{*}-\bar{Y} & ≅ & \sum_{h=1}^{L}P_{h}[\left(m_{1h}-1\right){\bar{Y}}_{h}+m_{1h}(W_{hY}+e_{h}t_{h}R_{h}W_{hX}+\frac{e_{h}t_{h}R_{h}W_{hX}W_{hY}}{\bar{X_{h}}}\\ & & +\frac{f_{h}R_{h}t_{h}^{2}W_{hX}^{2}}{{\bar{X}}_{h}})+m_{2h}\left(t_{h}W_{hX}+\frac{e_{h}t_{h}^{2}W_{hX}^{2}}{\bar{X_{h}}}\right)], \end{array}$$ (15)

where, $e_{h}=\frac{1+\alpha_{h}}{2}$ and $f_{h}=\frac{\alpha_{h}^{2}+4\alpha_{h}+3}{8}.$

Using Eq (15), the bias of ${\bar{y}}_{S(P)}^{*}$ to first order of approximation, is given by

$$\begin{array}{ccc} B({\bar{y}}_{S(P)}^{*}) & ≅ & \sum_{h=1}^{L}P_{h}[\left(m_{1h}-1\right){\bar{Y}}_{h}+m_{1h}\left(\frac{e_{h}t_{h}R_{h}C_{h}}{{\bar{X}}_{h}}+\frac{f_{h}R_{h}t_{h}^{2}B_{h}}{{\bar{X}}_{h}}\right)\\ & & +m_{2h}\left(\frac{e_{h}t_{h}^{2}B_{h}}{{\bar{X}}_{h}}\right)]. \end{array}$$ (16)

Squaring both sides of Eq (15), and keeping the terms up to power two in errors, and then taking expectations, the mean square error of ${\bar{y}}_{S(P)}^{*}$ is given by

$$\begin{array}{ccc} MSE({\bar{y}}_{S(P)}^{*}) & ≅ & \sum_{h=1}^{L}{P_{h}}^{2}[{\bar{Y}}_{h}^{2}+2m_{1h}m_{2h}\left(t_{h}C_{h}+2e_{h}t_{h}^{2}R_{h}B_{h}\right)\\ & & +m_{1h}^{2}\left({\bar{Y}}_{h}^{2}+A_{h}+e_{h}^{2}t_{h}^{2}R_{h}^{2}B_{h}+4e_{h}t_{h}R_{h}C_{h}+2f_{h}t_{h}^{2}R_{h}^{2}B_{h}\right)+m_{2h}^{2}t_{h}^{2}B_{h}\\ & & -2m_{1h}\left({\bar{Y}}_{h}^{2}+e_{h}t_{h}R_{h}C_{h}+f_{h}t_{h}^{2}R_{h}^{2}B_{h}\right)-2m_{2h}e_{h}t_{h}^{2}R_{h}B_{h}]. \end{array}$$

The above equation can be written as

$$\begin{array}{c} MSE\left({\bar{y}}_{S(P)}^{*}\right)≅\sum_{h=1}^{L}P_{h}^{2}\left[{\bar{Y}}_{h}^{2}+m_{1h}^{2}A_{h1}+m_{2h}^{2}B_{h1}+2m_{1h}m_{2h}C_{h1}-2m_{1h}D_{h1}-2m_{2h}E_{h1}\right], \end{array}$$ (17)

where, $A_{h1}={\bar{Y}}_{h}^{2}+A_{h}+e_{h}^{2}t_{h}^{2}R_{h}^{2}B_{h}+4e_{h}t_{h}R_{h}C_{h}+2f_{h}t_{h}^{2}R_{h}^{2}B_{h}$, $B_{h1}=t_{h}^{2}B_{h}$, $C_{h1}=t_{h}C_{h}+2e_{h}t_{h}^{2}R_{h}B_{h}$, $D_{h1}={\bar{Y}}_{h}^{2}+e_{h}t_{h}R_{h}C_{h}+f_{h}t_{h}^{2}R_{h}^{2}B_{h}$, $E_{h1}=e_{h}t_{h}^{2}R_{h}B_{h}$.

For finding the optimal values of *m*_1*h*_ and *m*_2*h*_, we differentiate Eq (17) with respect to *m*_1*h*_ and *m*_2*h*_ respectively. The optimal values are given by $m_{1h(opt)}=\frac{E_{h1}C_{h1}-D_{h1}B_{h1}}{C_{h1}^{2}-A_{h1}B_{h1}}$ and $m_{2h(opt)}=\frac{D_{h1}C_{h1}-A_{h1}E_{h1}}{C_{h1}^{2}-A_{h1}B_{h1}}$.

Substituting these optimum values in Eq (17), we get the minimum mean square error of ${\bar{y}}_{S(P)}^{*}$, as

$$\begin{array}{c} MSE{\left({\bar{y}}_{S(P)}^{*}\right)}_{min}≅\sum_{h=1}^{L}P_{h}^{2}\left[{\bar{Y}}_{h}^{2}-\frac{A_{h1}E_{h1}^{2}+B_{h1}D_{h1}^{2}-2C_{h1}D_{h1}E_{h1}}{\left(A_{h1}B_{h1}-C_{h1}^{2}\right)}\right]. \end{array}$$ (18)

## Efficiency comparison

The efficiency comparison of ${\bar{y}}_{S(HH)}^{*},{\bar{y}}_{S(R)}^{*},{\bar{y}}_{S(D)}^{*}$ and ${\bar{y}}_{S(AH)}^{*}$ with respect to ${\bar{y}}_{S(P)}^{*}$ are given by,

1. From Eqs (2) and (18), $MSE{\left({\bar{y}}_{S(P)}^{*}\right)}_{min}<Var\left({\bar{y}}_{S(HH)}^{*}\right)$ if $\sum_{h=1}^{L}P_{h}^{2}{\bar{Y_{h}}}^{2}-\sum_{h=1}^{L}{P_{h}}^{2}\left[\frac{A_{h1}E_{h1}^{2}+B_{h1}D_{h1}^{2}-2C_{h1}D_{h1}E_{h1}}{\left(A_{h1}B_{h1}-C_{h1}^{2}\right)}\right]-\sum_{h=1}^{L}{P_{h}}^{2}A_{h}<0$
2. From Eqs (5) and (18), $MSE{\left({\bar{y}}_{S(P)}^{*}\right)}_{min}<MSE\left({\bar{y}}_{S(R)}^{*}\right)$ if $\sum_{h=1}^{L}P_{h}^{2}{\bar{Y_{h}}}^{2}-\sum_{h=1}^{L}{P_{h}}^{2}\left[\frac{A_{h1}E_{h1}^{2}+B_{h1}D_{h1}^{2}-2C_{h1}D_{h1}E_{h1}}{\left(A_{h1}B_{h1}-C_{h1}^{2}\right)}\right]-\sum_{h=1}^{L}{P_{h}}^{2}\left[A_{h}+R_{h}^{2}B_{h}-2R_{h}C_{h}\right]<0$
3. From Eqs (7) and (18), $MSE{\left({\bar{y}}_{S(P)}^{*}\right)}_{min}<Var{\left({\bar{y}}_{S(D)}^{*}\right)}_{min}$ if $\sum_{h=1}^{L}P_{h}^{2}{\bar{Y_{h}}}^{2}-\sum_{h=1}^{L}{P_{h}}^{2}\left[\frac{A_{h1}E_{h1}^{2}+B_{h1}D_{h1}^{2}-2C_{h1}D_{h1}E_{h1}}{\left(A_{h1}B_{h1}-C_{h1}^{2}\right)}\right]-\sum_{h=1}^{L}{P_{h}}^{2}\left[A_{h}-\frac{C_{h}^{2}}{B_{h}}\right]<0$
4. From Eqs (10) and (18), $MSE{\left({\bar{y}}_{S(P)}^{*}\right)}_{min}<MSE\left({\bar{y}}_{S(AH)}^{*}\right)$ if $\sum_{h=1}^{L}P_{h}^{2}{\bar{Y_{h}}}^{2}-\sum_{h=1}^{L}{P_{h}}^{2}\left[\frac{A_{h1}E_{h1}^{2}+B_{h1}D_{h1}^{2}-2C_{h1}D_{h1}E_{h1}}{\left(A_{h1}B_{h1}-C_{h1}^{2}\right)}\right]-\sum_{h=1}^{L}{P_{h}}^{2}\left[A_{h}+q_{h}^{2}R_{h}^{2}B_{h}-2q_{h}R_{h}C_{h}\right]<0$

The proposed class of estimators is more efficient than other existing estimators when above conditions 1 to 4 are satisfied.

## Numerical results

In this section three populations are generated for simulation study and four are based on real data sets. The results are given in Tables 1–3 (simulation) and 4–7 (real data).

**Table 1:** Mean squared error (MSE) of different estimators for Pop I(*N* = 4000).

Estimators	10% non-response			20% non-response		
	*g*_*h*_			*g*_*h*_		
	2	4	8	2	4	8
${\bar{y}}_{HH}^{*}$	0.095391	0.116525	0.158794	0.105952	0.148191	0.232694
${\bar{y}}_{S(R)}^{*}$	0.021870	0.026292	0.035128	0.024495	0.034155	0.053486
${\bar{y}}_{S(D)}^{*}$	0.020585	0.024792	0.033190	0.023020	0.032094	0.050240
${\bar{y}}_{S(AH)}^{*}$	0.056410	0.068214	0.091802	0.063076	0.088209	0.138450
$\alpha =0,{\bar{y}}_{S(P)}^{*}$	**0.020450**	0.024598	0.032852	0.022853	0.031779	0.049480
$\alpha =1,{\bar{y}}_{S(P)}^{*}$	0.020458	0.024609	0.032873	0.022862	0.031797	0.049526
$\alpha =-1,{\bar{y}}_{S(P)}^{*}$	0.020459	0.024610	0.032874	0.022863	0.031798	0.049529

**Table 2:** Mean squared error (MSE) of different estimators for Pop II(*N* = 5000).

Estimators	10% non-response			20% non-response		
	*g*_*h*_			*g*_*h*_		
	2	4	8	2	4	8
${\bar{y}}_{HH}^{*}$	0.094074	0.114968	0.156755	0.102687	0.140807	0.217047
${\bar{y}}_{S(R)}^{*}$	0.012512	0.015799	0.022374	0.014212	0.020899	0.034275
${\bar{y}}_{S(D)}^{*}$	0.012504	0.015776	0.022302	0.014193	0.020816	0.034010
${\bar{y}}_{S(AH)}^{*}$	0.027283	0.034279	0.048270	0.030751	0.044685	0.072551
$\alpha =0,{\bar{y}}_{S(P)}^{*}$	**0.012463**	0.015712	0.022175	0.014141	0.020707	0.033723
$\alpha =1,{\bar{y}}_{S(P)}^{*}$	0.012466	0.015716	0.0221825	0.014144	0.020713	0.033738
$\alpha =-1,{\bar{y}}_{S(P)}^{*}$	0.012467	0.015717	0.0221827	0.014145	0.020714	0.033739

**Table 3:** Mean squared error (MSE) of different estimators for Pop III(*N* = 800).

Estimators	10% non-response			20% non-response		
	*g*_*h*_			*g*_*h*_		
	2	4	8	2	4	8
${\bar{y}}_{HH}^{*}$	0.905181	1.034025	1.291698	1.004663	1.332462	1.988063
${\bar{y}}_{S(R)}^{*}$	0.280667	0.331342	0.432703	0.302164	0.395841	0.583195
${\bar{y}}_{S(D)}^{*}$	0.199208	0.232876	0.299860	0.212367	0.272204	0.391440
${\bar{y}}_{S(AH)}^{*}$	0.559174	0.655443	0.847981	0.614328	0.820925	1.234103
$\alpha =0,{\bar{y}}_{S(P)}^{*}$	**0.193840**	0.225546	0.287533	0.206202	0.261988	0.370120
$\alpha =1,{\bar{y}}_{S(P)}^{*}$	0.193986	0.225734	0.287820	0.206378	0.262264	0.370686
$\alpha =-1,{\bar{y}}_{S(P)}^{*}$	0.194009	0.225766	0.287885	0.206396	0.262314	0.370836

**Table 4:** Mean squared error (MSE) of different estimators for Pop IV(N = 854).

Estimators	10% non-response			20% non-response		
	*g*_*h*_			*g*_*h*_		
	2	4	8	2	4	8
${\bar{y}}_{HH}^{*}$	2719483	3340426	4582312	3025218	4257631	6722457
${\bar{y}}_{S(R)}^{*}$	435763.4	536225.8	737150.5	470462.5	640323.1	980044.2
${\bar{y}}_{S(D)}^{*}$	278381.2	341063.4	466350.4	298383.2	400062.1	601701.3
${\bar{y}}_{S(AH)}^{*}$	293568.4	360869.7	495472.4	316102.2	428471.3	653209.4
$\alpha =0,{\bar{y}}_{S(P)}^{*}$	**232820.0**	276450.6	356353.0	247489.8	316893.5	438483.9
$\alpha =1,{\bar{y}}_{S(P)}^{*}$	233140.6	276830	356763.9	247852.6	317379.8	438961.3
$\alpha =-1,{\bar{y}}_{S(P)}^{*}$	233433.5	277328.6	357873.8	248211.2	318171.9	441257.5

**Table 5:** Mean squared error (MSE) of different estimators for Pop V(N = 120).

Estimators	10% non-response			20% non-response		
	*g*_*h*_			*g*_*h*_		
	2	4	8	2	4	8
${\bar{y}}_{HH}^{*}$	219.5693	280.1731	401.3808	254.9066	386.1849	648.7416
${\bar{y}}_{S(R)}^{*}$	311.3524	395.5183	563.8503	304.1427	373.8894	513.3828
${\bar{y}}_{S(D)}^{*}$	191.9500	243.8635	347.6900	218.4376	316.3906	489.3592
${\bar{y}}_{S(AH)}^{*}$	1041.7910	1330.7270	1908.6000	932.9254	1004.1320	1146.5440
$\alpha =0,{\bar{y}}_{S(P)}^{*}$	**130.7954**	149.6804	174.9338	143.8646	181.0074	225.6080
$\alpha =1,{\bar{y}}_{S(P)}^{*}$	132.0265	154.0230	187.7458	145.5440	185.1315	235.0778
$\alpha =-1,{\bar{y}}_{S(P)}^{*}$	137.6204	162.4859	203.0227	151.0114	192.6844	246.5152

**Table 6:** Mean squared error (MSE) of different estimators for Pop VI(N = 120).

Estimators	10% non-response			20% non-response		
	*g*_*h*_			*g*_*h*_		
	2	4	8	2	4	8
${\bar{y}}_{HH}^{*}$	249.1903	369.0362	608.728	254.906	386.1843	648.741
${\bar{y}}_{S(R)}^{*}$	0.400780	0.594000	0.980242	1.921332	5.155446	11.623670
${\bar{y}}_{S(D)}^{*}$	0.354179	0.5248469	0.8659781	1.79562	4.655165	10.13164
${\bar{y}}_{S(AH)}^{*}$	352.2648	522.7970	863.8607	363.5492	556.6457	942.8387
$\alpha =0,{\bar{y}}_{S(P)}^{*}$	**0.103377**	0.468480	0.524870	1.476885	3.736331	7.129239
$\alpha =1,{\bar{y}}_{S(P)}^{*}$	0.353887	0.495700	0.723897	1.772095	4.481196	9.319902
$\alpha =-1,{\bar{y}}_{S(P)}^{*}$	0.353936	0.494876	0.725973	1.773337	4.493499	9.413715

**Table 7:** Mean squared error (MSE) of different estimators for Pop VII(N = 119).

Estimators	10% non-response			20% non-response		
	*g*_*h*_			*g*_*h*_		
	2	4	8	2	4	8
${\bar{y}}_{HH}^{*}$	630.3935	805.3869	1155.3740	713.2246	1053.8800	1735.1910
${\bar{y}}_{S(R)}^{*}$	55.4937	70.4183	100.2675	61.3649	88.0319	141.3660
${\bar{y}}_{S(D)}^{*}$	54.9625	69.7884	99.4359	60.7277	87.0779	139.7685
${\bar{y}}_{S(AH)}^{*}$	508.7321	652.4944	940.0190	574.8255	850.7745	1402.6730
$\alpha =0,{\bar{y}}_{S(P)}^{*}$	**54.7160**	69.3872	98.6146	60.4094	86.3789	137.8763
$\alpha =1,{\bar{y}}_{S(P)}^{*}$	54.7836	69.4961	98.8344	60.4953	86.5623	138.3569
$\alpha =-1,{\bar{y}}_{S(P)}^{*}$	54.7847	69.4983	98.8410	60.4969	86.5677	138.3814

### Simulation study

We have generated three populations from normal distribution by using R language program. First population is generated for equal strata and the second one is generated for unequal strata and third one is generated for equal strata of small sample size (see Appendix).

The above tables shows that a general class of proposed estimators outperform all the other existing estimators. For $\alpha =0,{\bar{y}}_{S(P)}^{*}$ shows the better performance.

### Application to real data

In this section we have considered four data sets (see Appendix) for numerical comparisons and results are given in Tables 4–7.

In these tables, we observed that a general class of proposed estimators is more efficient as compared to all other considered estimators. For $\alpha =0,{\bar{y}}_{S(P)}^{*}$ shows the better performance.

## Conclusion

In the present paper, we have suggested an improved class of estimators of the finite population mean in the presence of measurement error and non-response under stratified random sampling. Through simulation study and real life data sets it is observed that the proposed class of estimators perform better than the existing estimators. The mean square error values are generally smaller under 10% of non-response as compared to 20% of non-response, which are expected results. Generally as the non-response rate increases, mean square error also increases. Based on numerical findings, it turns out that the proposed class of estimators is more efficient for the situations when *α* = 0, *α* = 1 and *α* = −1 as compared to the other existing estimators. Among different classes, the performance of proposed class of estimators is better for *α* = 0 in Tables 1–7.

## Appendix

**Population I.**

*X*_1_ = *rnorm*(1000, 5, 10), *Y*_1_ = *X*_1_ + *rnorm*(1000, 0, 1), *y*_1_ = *Y*_1_ + *rnorm*(1000, 1, 3), *x*_1_ = *X*_1_ + *rnorm*(1000, 1, 3)

*X*_2_ = *rnorm*(1000, 4, 8), *Y*_2_ = *X*_2_ + *rnorm*(1000, 0, 1), *y*_2_ = *Y*_2_ + *rnorm*(1000, 1, 3), *x*_2_ = *X*_2_ + *rnorm*(1000, 1, 3)

*X*_3_ = *rnorm*(1000, 4, 9), *Y*_3_ = *X*_3_ + *rnorm*(1000, 0, 1), *y*_3_ = *Y*_3_ + *rnorm*(1000, 1, 3), *x*_3_ = *X*_3_ + *rnorm*(1000, 1, 3)

*X*_4_ = *rnorm*(1000, 3, 7), *Y*_4_ = *X*_4_ + *rnorm*(1000, 0, 1), *y*_4_ = *Y*_4_ + *rnorm*(1000, 1, 3), *x*_4_ = *X*_4_ + *rnorm*(1000, 1, 3)

*Number of Stratas* = 4

*N*_1_ = 1000, *N*_2_ = 1000, *N*_3_ = 1000, *N*_4_ = 1000, *n*_1_ = 200, *n*_2_ = 200, *n*_3_ = 200, *n*_4_ = 200, ${\bar{Y}}_{1}=5.670898$, ${\bar{Y}}_{2}=3.646459$, ${\bar{Y}}_{3}=4.039068$, ${\bar{Y}}_{4}=2.902825$, ${\bar{X}}_{1}=5.666893$, ${\bar{X}}_{2}=3.643237$, ${\bar{X}}_{3}=3.968049$, ${\bar{X}}_{4}=2.918596$, $S_{1Y}^{2}=106.310$, $S_{2Y}^{2}=67.69053$, $S_{3Y}^{2}=81.46952$, $S_{4Y}^{2}=47.2278$, $S_{1X}^{2}=104.2774$, $S_{2X}^{2}=66.19725$, $S_{3X}^{2}=81.06883$, $S_{4X}^{2}=45.99937$, *ρ*_1*YX*_ = 0.9950779, *ρ*_2*YX*_ = 0.9926346, *ρ*_3*YX*_ = 0.9939164, *ρ*_4*YX*_ = 0.9896319.

**Population II.**

*X*_1_ = *rnorm*(1000, 5, 10), *Y*_1_ = *X*_1_ + *rnorm*(1000, 0, 1), *y*_1_ = *Y*_1_ + *rnorm*(1000, 1, 3), *x*_1_ = *X*_1_ + *rnorm*(1000, 0, 1)

*X*_2_ = *rnorm*(1200, 4, 8), *Y*_2_ = *X*_2_ + *rnorm*(1200, 0, 1), *y*_2_ = *Y*_2_ + *rnorm*(1200, 1, 3), *x*_2_ = *X*_2_ + *rnorm*(1200, 0, 1)

*X*_3_ = *rnorm*(1300, 4, 9), *Y*_3_ = *X*_3_ + *rnorm*(1300, 0, 1), *y*_3_ = *Y*_3_ + *rnorm*(1300, 1, 3), *x*_3_ = *X*_3_ + *rnorm*(1300, 0, 1)

*X*_4_ = *rnorm*(1500, 3, 7), *Y*_4_ = *X*_4_ + *rnorm*(1500, 0, 1), *y*_4_ = *Y*_4_ + *rnorm*(1500, 1, 3), *x*_4_ = *X*_4_ + *rnorm*(1500, 1, 3)

*Number of Stratas* = 4

*N*_1_ = 1000, *N*_2_ = 1200, *N*_3_ = 1300, *N*_4_ = 1500, *n*_1_ = 200, *n*_2_ = 210, *n*_3_ = 220, *n*_4_ = 215, ${\bar{Y}}_{1}=5.670898$, ${\bar{Y}}_{2}=3.79172$, ${\bar{Y}}_{3}=4.582486$, ${\bar{Y}}_{4}=3.20716$, ${\bar{X}}_{1}=5.666893$, ${\bar{X}}_{2}=3.807569$, ${\bar{X}}_{3}=4.627208$, ${\bar{X}}_{4}=3.241139$, $S_{1Y}^{2}=106.3107$, $S_{2Y}^{2}=66.3915$, $S_{3Y}^{2}=84.23239$, $S_{4Y}^{2}=53.52266$, $S_{1X}^{2}=104.2774$, $S_{2X}^{2}=65.46337$, $S_{3X}^{2}=82.98812$, $S_{4X}^{2}=52.84269$, *ρ*_1*YX*_ = 0.9950779, *ρ*_2*YX*_ = 0.9924618, *ρ*_3*YX*_ = 0.9939475, *ρ*_4*YX*_ = 0.9903624.

**Population III.**

*X*_1_ = *rnorm*(200, 5, 10), *Y*_1_ = *X*_1_ + *rnorm*(200, 0, 1), *y*_1_ = *Y*_1_ + *rnorm*(200, 1, 3), *x*_1_ = *X*_1_ + *rnorm*(200, 0, 1)

*X*_2_ = *rnorm*(200, 4, 8), *Y*_2_ = *X*_2_ + *rnorm*(200, 0, 1), *y*_2_ = *Y*_2_ + *rnorm*(200, 1, 3), *x*_2_ = *X*_2_ + *rnorm*(1200, 0, 1)

*X*_3_ = *rnorm*(200, 4, 9), *Y*_3_ = *X*_3_ + *rnorm*(200, 0, 1), *y*_3_ = *Y*_3_ + *rnorm*(200, 1, 3), *x*_3_ = *X*_3_ + *rnorm*(1300, 0, 1)

*X*_4_ = *rnorm*(200, 3, 7), *Y*_4_ = *X*_4_ + *rnorm*(200, 0, 1), *y*_4_ = *Y*_4_ + *rnorm*(200, 1, 3), *x*_4_ = *X*_4_ + *rnorm*(200, 1, 3)

*Number of Stratas* = 4

*N*_1_ = 200, *N*_2_ = 200, *N*_3_ = 200, *N*_4_ = 200, *n*_1_ = 22, *n*_2_ = 28, *n*_3_ = 22, *n*_4_ = 25, ${\bar{Y}}_{1}=7.470477$, ${\bar{Y}}_{2}=4.815927$, ${\bar{Y}}_{3}=5.206734$, ${\bar{Y}}_{4}=4.864139$, ${\bar{X}}_{1}=5.895905$, ${\bar{X}}_{2}=3.720469$, ${\bar{X}}_{3}=3.9945$, ${\bar{X}}_{4}=3.698503$, $S_{1Y}^{2}=113.6437$, $S_{2Y}^{2}=73.19005$, $S_{3Y}^{2}=92.51202$, $S_{4Y}^{2}=62.38946$, $S_{1X}^{2}=105.978$, $S_{2X}^{2}=68.09821$, $S_{3X}^{2}=89.87238$, $S_{4X}^{2}=46.12838$, *ρ*_1*YX*_ = 0.9447295, *ρ*_2*YX*_ = 0.9214058, *ρ*_3*YX*_ = 0.9529337, *ρ*_4*YX*_ = 0.912232.

**Population IV.** (Source: [18])

**Y:** Number of teachers, **X:** Number of students.

*Number of Stratas* = 6

*N*_1_ = 106, *N*_2_ = 106, *N*_3_ = 94, *N*_4_ = 171, *N*_5_ = 204, *N*_6_ = 173, *n*_1_ = 15, *n*_2_ = 15, *n*_3_ = 12, *n*_4_ = 20, *n*_5_ = 23, *n*_6_ = 15, ${\bar{Y}}_{1}=1536.774$, ${\bar{Y}}_{2}=2212.594$, ${\bar{Y}}_{3}=9384.309$, ${\bar{Y}}_{4}=5588.012$, ${\bar{Y}}_{5}=966.9559$, ${\bar{Y}}_{6}=404.3988$, ${\bar{X}}_{1}=24375.59$, ${\bar{X}}_{2}=27421.70$, ${\bar{X}}_{3}=72409.95$, ${\bar{X}}_{4}=74364.68$, ${\bar{X}}_{5}=26441.72$, ${\bar{X}}_{6}=9842.15$, $S_{1Y}^{2}=41281746$, $S_{2Y}^{2}=133437791$, $S_{3Y}^{2}=894457433$, $S_{4Y}^{2}=820445636$, $S_{5Y}^{2}=5710999$, $S_{6Y}^{2}=894440.3$, $S_{1X}^{2}=2419565835$, $S_{2X}^{2}=3301722268$, $S_{3X}^{2}=25842911895$, $S_{4X}^{2}=81569146488$, $S_{5X}^{2}=2061412416$, $S_{6X}^{2}=353245374$. *ρ*_1*YX*_ = 0.8156414, *ρ*_2*YX*_ = 0.8156414, *ρ*_3*YX*_ = 0.9011201, *ρ*_4*YX*_ = 0.9858761, *ρ*_5*YX*_ = 0.7130988, *ρ*_6*YX*_ = 0.893595.

**Population V.** (Source: [19])

**Y:** 1983 population(in millions), **X:** 1982 gross national product

*Number of Stratas* = 5

*N*_1_ = 38, *N*_2_ = 14, *N*_3_ = 11, *N*_4_ = 33, *N*_5_ = 24, *n*_1_ = 17, *n*_2_ = 6, *n*_3_ = 4, *n*_4_ = 12, *n*_5_ = 11, ${\bar{Y}}_{1}=13.03684$, ${\bar{Y}}_{2}=27.35$, ${\bar{Y}}_{3}=23.13636$, ${\bar{Y}}_{4}=79.65455$, ${\bar{Y}}_{5}=20.28333$, ${\bar{X}}_{1}=1029.158$, ${\bar{X}}_{2}=25671.57$, ${\bar{X}}_{3}=5028.818$, ${\bar{X}}_{4}=7533.939$, ${\bar{X}}_{5}=16315.25$, $S_{1Y}^{2}=270.9083$, $S_{2Y}^{2}=3906.929$, $S_{3Y}^{2}=1339.405$, $S_{4Y}^{2}=45082.17$, $S_{5Y}^{2}=368.9423$, $S_{1X}^{2}=3667896$, $S_{2X}^{2}=6568461403$, $S_{3X}^{2}=63348743$, $S_{4X}^{2}=440717912$, $S_{5X}^{2}=408441212$, *ρ*_1*YX*_ = 0.7439544, *ρ*_2*YX*_ = 0.969956, *ρ*_3*YX*_ = 0.9768227, *ρ*_4*YX*_ = 0.2948897, *ρ*_5*YX*_ = 0.9011072.

**Population VI.** (Source: [19])

**Y:** 1983 population(in millions), **X:** 1980 population(in millions)

*Number of Stratas* = 5

*N*_1_ = 38, *N*_2_ = 14, *N*_3_ = 11, *N*_4_ = 33, *N*_5_ = 24, *n*_1_ = 17, *n*_2_ = 6, *n*_3_ = 4, *n*_4_ = 12, *n*_5_ = 11, ${\bar{Y}}_{1}=13.03684$, ${\bar{Y}}_{2}=27.35$, ${\bar{Y}}_{3}=23.13636$, ${\bar{Y}}_{4}=79.65455$, ${\bar{Y}}_{5}=20.28333$, ${\bar{X}}_{1}=11.88421$, ${\bar{X}}_{2}=26.18571$, ${\bar{X}}_{3}=21.88182$, ${\bar{X}}_{4}=75.24242$, ${\bar{X}}_{5}=20.09583$, $S_{1Y}^{2}=270.9083$, $S_{2Y}^{2}=3906.929$, $S_{3Y}^{2}=1339.405$, $S_{4Y}^{2}=45082.17$, $S_{5Y}^{2}=368.9423$, $S_{1X}^{2}=222.4889$, $S_{2X}^{2}=3683.071$, $S_{3X}^{2}=1174.032$, $S_{4X}^{2}=41280.19$, $S_{5X}^{2}=364.7839$, *ρ*_1*YX*_ = 0.9996193, *ρ*_2*YX*_ = 0.9998693, *ρ*_3*YX*_ = 0.9998858, *ρ*_4*YX*_ = 0.9993071, *ρ*_5*YX*_ = 0.9998059.

**Population VII.** ([20])

**Y:** Production of wheat(in tons), **X:** Area of wheat (in hectares)

*Number of Stratas* = 4

*N*_1_ = 47, *N*_2_ = 30, *N*_3_ = 29, *N*_4_ = 13, *n*_1_ = 15, *n*_2_ = 10, *n*_3_ = 10, *n*_4_ = 5, $\bar{Y_{1}}=443.5447$, $\bar{Y_{2}}=68.68276$, $\bar{Y_{3}}=17.06667$, $\bar{Y_{4}}=52.52308$, $\bar{X_{1}}=160.2362$, $\bar{X_{2}}=29.70345$, $\bar{X_{3}}=11.54667$, $\bar{X_{4}}=23.62308$, $S_{1Y}^{2}=74026.75$, $S_{2Y}^{2}=2871.781$, $S_{3Y}^{2}=244.1292$, $S_{4Y}^{2}=4451.124$, $S_{1X}^{2}=8377.401$, $S_{2X}^{2}=316.4532$, $S_{3X}^{2}=91.45775$, $S_{4X}^{2}=682.9703$, *ρ*_1*YX*_ = 0.9583838, *ρ*_2*YX*_ = 0.779071, *ρ*_3*YX*_ = 0.8719665, *ρ*_4*YX*_ = 0.9922591.

## Supporting information

S1 File Data used in the manuscript “S1 File.zip”. (ZIP)
